# rAAV Vectors as Safe and Efficient Tools for the Stable Delivery of Genes to Primary Human Chondrosarcoma Cells *In Vitro* and *In Situ*

**DOI:** 10.1155/2012/347417

**Published:** 2012-05-07

**Authors:** Henning Madry, Jagadeesh K. Venkatesan, Gertrud Schmitt, Sarah Schetting, Myriam Ekici, Dieter Kohn, Magali Cucchiarini

**Affiliations:** ^1^Center of Experimental Orthopaedics, Saarland University Medical Center, Saarland, 66421 Homburg, Germany; ^2^Department of Orthopaedic Surgery, Saarland University Medical Center, Saarland, 66421 Homburg, Germany

## Abstract

Treatment of chondrosarcoma remains a major challenge in orthopaedic oncology. Gene transfer strategies based on recombinant adenoassociated viral (rAAV) vectors may provide powerful tools to develop new, efficient therapeutic options against these tumors. In the present study, we tested the hypothesis that rAAV is adapted for a stable and safe delivery of foreign sequences in human chondrosarcoma tissue by transducing primary human chondrosarcoma cells *in vitro* and *in situ* with different reporter genes (*E. coli lacZ*, firefly *luc, Discosoma sp.* RFP). The effects of rAAV administration upon cell survival and metabolic activities were also evaluated to monitor possibly detrimental effects of the gene transfer method. Remarkably, we provide evidence that efficient and prolonged expression of transgene sequences via rAAV can be safely achieved in all the systems investigated, demonstrating the potential of the approach of direct application of therapeutic gene vectors as a means to treat chondrosarcoma.

## 1. Introduction

Chondrosarcomas are a complex group of primary solid cartilaginous tumors with variable clinical behavior and histopathology. They are classified as either central (skeletal) chondrosarcomas, including conventional, dedifferentiated, mesenchymal, or of clear cell subtype, or peripheral (extraskeletal) chondrosarcomas of myxoid type, from solitary osteochondromas, or associated with the hereditary multiple exostoses syndrome. These differences are reflected by the diversity of genetic abnormalities observed (chromosomal translocations, rearrangements, duplications, deletions) [1–4]. Among them, the conventional subtypes that are usually assessed according to clinicoradiologic and histopathological criteria from grade 1 to 3 [5–9] represent about 90% of skeletal chondrosarcomas. Surgical management of these tumors in individuals is currently the only curative treatment, as chondrosarcomas do not respond well to radio- and/or chemotherapy, indicating a potential need for novel therapeutic approaches. 

Large efforts have been made to understand the mechanisms underlying the pathogenesis of these tumors [1, 4, 10–13]. Indeed, evidence has been provided showing the alteration of tumor suppressors (p53, retinoblastoma) and the activation of oncogenes (c-myc), signaling axes (Bcl-2, Ihh/PTHrP, GH/IGF, FGF-2/FGFR1, survivin), or angiogenic factors (VEGF, FGF-2). Such findings may allow to identify new targets for therapy in addition to those already involved in cell proliferative and cartilage-related synthetic pathways (overexpression of type-II and type-X collagen, aggrecan, fibronectin, some matrix metalloproteinases MMPs, SOX9, S-100) [5–9, 14–16].

Regarding the development of novel therapeutic approaches, delivery of candidate genes in chondrosarcoma tissue might be a powerful tool to generate efficient and durable treatments against chondrosarcoma in patients [17, 18]. Strategies with potential benefits against the progression of such tumors might be based on the application of either directly interfering genetic sequences (antisense/siRNA strategies, specific antagonists) or of genes coding for antitumor, antiangiogenic, proapoptotic, or antidifferentiative agents (herpes simplex thymidine kinase HSV-tk, p53, chondromodulin I, endostatin, oncostatin M OSM, some Wnts) [1, 4, 19–46]. So far, few studies have demonstrated the possibility of delivering genes in human chondrosarcoma cells and tissue, most of which being based on the use of nonviral [25, 26, 29, 30, 45–47] and classical viral vectors (adenoviral, retro-, and lentiviral vectors) [19, 27, 28, 32, 36, 40, 41] that exhibit relatively low gene transfer efficacies (and thus requiring the need of a complex cell selection prior to use as platforms for therapy: nonviral and retroviral vectors), induce immunogenic responses (adenoviral vectors), or carry the risk of insertional mutagenesis (retro- and lentiviral vectors). Protocols based on the use of vectors derived from the adenoassociated virus (AAV) might offer good alternatives as recombinant AAV (rAAV) are replication-defective human vectors that carry none of the AAV protein-coding sequences (making them less immunogenic than adenoviral vectors) and that are maintained and expressed as highly stable episomes [48, 49] (lowering the risk of insertional mutagenesis), making rAAV a currently favored gene transfer system for human clinical trials [50]. To date, and to our best knowledge, there is no evidence showing the possibility of targeting human chondrosarcoma tissue using rAAV as a gene transfer system. Therefore, in the present study we tested the ability of rAAV to efficiently and stably deliver different reporter genes in chondrosarcoma cells *in vitro* and most importantly *in situ* and further analyzed the potential damaging effects of the gene transfer procedure upon the activities of these cells in all systems evaluated.

## 2. Materials and Methods

### 2.1. Reagents

All reagents were from Sigma (Munich, Germany) except for the collagenase type I (232 U/mg) (Biochrom, Berlin, Germany). The anti-*β*-gal (GAL-13) and anti-type-X collagen (COL-10) antibodies were from Sigma (Munich, Germany), the anti-type-II collagen (AF-5710) and anti-type-I collagen (AF-5610) antibodies from Acris Antibodies GmbH (Herford, Germany), the anti-Ki-67 (PP-67) and anti-SOX9 (C-20) antibodies from Santa Cruz Biotechnology (Heidelberg, Germany), and the anti-S-100 (Z0311) antibody from Dako Deutschland GmbH (Hamburg, Germany). Luciferase activity was determined with the Luciferase Assay System (Promega GmbH, Mannheim, Germany) and normalized to total cellular proteins using the BCA protein assay kit (Pierce Thermo Scientific, Fisher Scientific GmbH, Schwerte, Germany). The cell proliferation reagent WST-1 and the Cytotoxicity Detection Kit (LDH) were from Roche Applied Science (Mannheim, Germany). Apoptosis was determined using the ApopTag Plus Peroxidase *In Situ* Apoptosis Detection Kit (Chemicon-Millipore GmbH, Schwalbach, Germany).

### 2.2. Tissue and Cells

Human chondrosarcoma tissue was obtained from patients undergoing tumor surgery (*n* = 6) (all chondrosarcoma graded 1 by an experienced pathologist of the Saarland University Medical Center on part of histological sections) [5–9]. All patients provided informed consent prior to inclusion in the study. For cell isolation, explants were washed, digested in collagenase [51], and resuspended in DMEM with 100 U/mL penicillin G and 100 *μ*l/mL streptomycin (basal medium). The cells were filtered through a 125 *μ*m mesh to remove the undigested matrix, and the cell numbers were determined by hemocytometry. Viability, as determined by trypan blue exclusion, exceeded 90% in all experiments. Cells were further maintained in basal medium containing 10% FBS (growth medium) at 37°C in a humidified atmosphere with 5% CO_2_. All experiments were performed with cells at not more than passage 1-2. For the experiments *in situ*, some explants were kept in growth medium at 37°C in a humidified atmosphere with 5% CO_2_. The 293 line, an adenovirus-transformed human embryonic kidney cell line, was maintained in Eagle's minimal essential medium containing 10% FBS and antibiotics.

### 2.3. Plasmids and rAAV Vectors

The constructs used in this study were derived from a parental AAV-2 genomic clone, pSSV9 [52, 53]. pAd8 contains the AAV-2 replication and encapsidation functions [53]. rAAV-*lacZ* is an AAV-2-based vector plasmid carrying the *lacZ* gene encoding *β*-galactosidase (*β*-gal) under the control of the cytomegalovirus immediate-early (CMV-IE) promoter [54–61]. rAAV-RFP carries a 776 bp *Discosoma* sp. red fluorescent protein (RFP) cDNA fragment and rAAV-*luc* carries the Firefly luciferase (*luc*) cDNA (1.7 kb) from pSP*luc*+ (Promega GmbH) that were cloned in rAAV-*lacZ* instead of the *lacZ* sequence [54, 56, 57, 61]. rAAV vectors were packaged as conventional (not self-complementary) elements using adenovirus 5 to provide helper functions in combination with pAd8, and the vector preparations were purified by dialysis and titered by real-time PCR [54–61], averaging 10^10^ units/mL (ratio of virus particles to functional vectors = 500/1) [56].

### 2.4. rAAV Transduction

Monolayer cultures of human chondrosarcoma cells (2 × 10^4^ cells) were transduced with rAAV (20 or 40 *μ*L each vector; MOI = 20–40) as previously described [54–61] and kept in culture for up to 20 days. *In situ*, the vectors (40 *μ*L each) were directly and homogeneously applied to various zones of the primary human chondrosarcoma explants that were also maintained in culture for up to 20 days.

### 2.5. Vector Copy Number Determination and Integration Site Analysis

Monolayer cultures of human chondrosarcoma cells (2 × 10^5^) were transduced with rAAV (200 *μ*l; MOI = 20) as described above or let untreated and kept in culture for up to 15 days. To determine the vector copy numbers, cells were processed using the QIAprep Spin Miniprep Kit (Qiagen GmbH, Hilden, Germany) to separate transgenic from genomic DNA according to the manufacturer's instructions. Vector copies were determined by real-time PCR using 500 ng DNA and the Brilliant SYBR Green QPCR Master Mix (Stratagene, Agilent Technologies GmbH, Waldbronn, Germany) on an Mx3000P QPCR operator system (Stratagene) to detect a region in the simian virus 40 small *t* antigen intron/polyadenylation signal present in the rAAV vectors and with a standard curve made of serially diluted plasmid containing the vector sequence at known concentrations, as previously described [54, 56, 62]. To determine potential AAV integration into chromosome 19, cells were processed using the QIAamp DNA Mini Kit (Qiagen) to isolate genomic DNA according to the manufacturer's instructions. Integration was detected by nested PCR using 100 ng DNA and primers flanking the AAV-chromosome 19 junction as previously described [63, 64].

### 2.6. Gene Transfer Analyses

Expression of the transgenes was determined by X-gal staining, live fluorescence, detection of luciferase activity normalized to total cellular proteins, and immunohistochemistry using specific primary antibodies and biotinylated secondary antibodies (Vector Laboratories, Alexis Deutschland GmbH, Grünberg, Germany) using the ABC method (Vector Laboratories) with diaminobenzidine (DAB) as the chromogen [54–61]. To control for secondary immunoglobulins, samples were processed with omission of the primary antibody. Samples were examined directly by light microscopy (Olympus BX 45; Hamburg, Germany) or by fluorescent microscopy using an Olympus microscope with a 568 nm filter (CKX41). Transduction efficiencies were calculated as previously described [54–61].

### 2.7. Histological and Immunohistochemical Analyses

Explants were histologically processed as previously described [57–59, 61]. Paraffin-embedded sections (5 *μ*m) were stained with hematoxylin and eosin (H&E) to detect cells or with safranin O to detect proteoglycans according to routine protocols [54, 55, 57–59, 61]. Fast green was used as a counterstain. Expression of Ki-67, type-II, type-I, and type-X collagen, SOX9, and S-100 was detected by immunohistochemistry using specific antibodies as previously described [5–8, 14–16, 55, 57–59].

### 2.8. Cell Survival, Death, and Cytotoxicity

The cell numbers and viability *in vitro* were determined by trypan blue exclusion over the course of the evaluation as previously described [54, 55, 57–59], and proliferation was assessed using the cell proliferation reagent WST-1 [54], with OD proportional to the cell numbers. Cell death by apoptosis *in vitro* was examined using the terminal deoxynucleotidyl transferase-mediated dUTP nick end labeling (TUNEL) method using the ApopTag Plus Peroxidase *In Situ* Apoptosis Detection Kit [36, 65]. Cytotoxicity *in vitro* was monitored using the Cytotoxicity Detection Kit (LDH) by measuring the release of lactate dehydrogenase (LDH) activity from damaged cells, with data given as percents of cytotoxicity *vis-à-vis* untransduced cells. *In situ*, cell proliferation was analyzed by immunodetection of the Ki-67 proliferation antigen [8] while cell death by apoptosis was assessed by TUNEL method (ApopTag Plus Peroxidase *In Situ* Apoptosis Detection Kit) [58].

### 2.9. Histomorphometric Analyses

The transduction efficiencies, the cell densities (H&E staining), the intensities of safranin O staining and those of type-II and type-I collagen immunostaining, the percents of cells positive for the expression of Ki-67, type-X collagen, SOX9, and S-100, and the percents of apoptotic cells were measured at 3 standardized sites using triplicate cultures or 10 serial sections per condition and time point using SIS AnalySIS (Olympus), Adobe Photoshop (Adobe Systems, Unterschleissheim, Germany), and Scion Image (Scion Corporation, Frederick, MD, USA) [54, 55, 57–59]. The percents of safranin O staining intensity were calculated as being the ratio of positively stained tissue surface to the total surface of the site evaluated. The type-II and type-I collagen immunostaining intensities were in pixels per standardized area.

### 2.10. Statistical Analysis

Data are expressed as mean ± standard deviation (SD) of separate experiments. Each condition was performed in triplicate in three independent experiments with monolayer and explant cultures. Data were obtained by two individuals that were blinded with respect to the treatment groups. The *t*-test and the Mann-Whitney rank sum test were employed where appropriate. Any *P* value of less than 0.05 was considered statistically significant.

## 3. Results

### 3.1. Efficient and Sustained rAAV-Mediated Gene Transfer in Primary Human Chondrosarcoma Cells *In Vitro*

**Figure 1:**
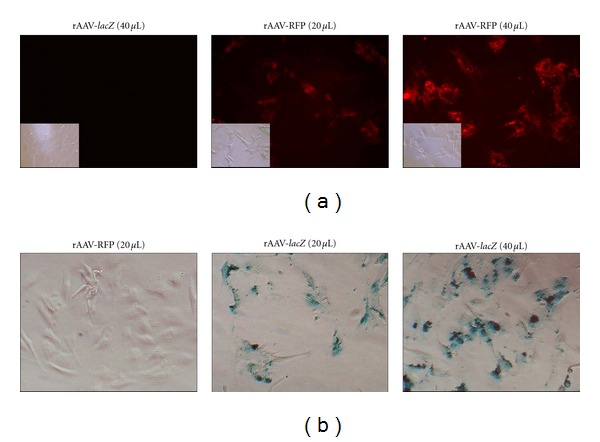
rAAV-mediated gene transfer in primary human chondrosarcoma cells *in vitro*. Cells in monolayer culture were transduced with rAAV-RFP or rAAV-*lacZ* (20 or 40 *μ*L) and processed to monitor transgene expression 20 days after vector application by analyzing live fluorescence ((a) magnification ×40; insets: same fields under transmitted light) and by X-gal staining ((b) magnification ×20).

Primary human chondrosarcoma cells were first transduced in monolayer culture using various reporter, control vectors (rAAV-RFP or rAAV-*lacZ*) to test the potentiality of rAAV to promote stable transgene expression in these cells *in vitro*. While a dose-dependent, prolonged (at least 20 days) fluorescent signal was noted only in cells transduced with rAAV-RFP (Figure 1(a)), X-gal staining was restricted to those where rAAV-*lacZ* was applied (Figure 1(b)). Remarkably, transduction efficiencies reached up to 82–90% at the highest vector dose applied (MOI = 40). In addition, administration of rAAV-*luc* to the cells at a higher vector dose also revealed continuously significant and sustained activities that were up to 25.6-fold higher compared with the control treatment (always *P* ≤ 0.001) (Table 1). When the cells were let untreated, no signal specific of any of the transgenes tested could be detected (data not shown). An analysis of the vector copy numbers in all cells transduced with rAAV (MOI = 20) revealed stable values ranging between 3 and 5 copies per cell from days 5 to 15, respectively, *versus* untreated cells. Also interestingly, no events of vector integration were detectable in all cultures tested at any time point of the analysis (data not shown).

### 3.2. Efficient and Sustained rAAV-Mediated Gene Transfer in Primary Human Chondrosarcoma Cells *In Situ*

**Figure 2:**
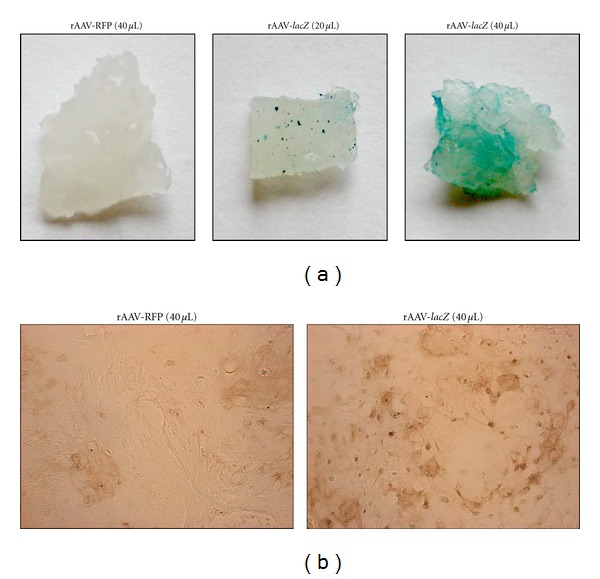
rAAV-mediated gene transfer in primary human chondrosarcoma *in situ*. Human chondrosarcoma explant cultures were transduced with rAAV-RFP or rAAV-*lacZ* (20 or 40 *μ*L) and processed to monitor transgene expression 20 days after vector application by X-Gal staining (a) and for immunodetection of *β*-gal activity ((b) magnification ×20).

The rAAV vectors were next provided to primary human chondrosarcoma tissue explants to further examine the ability of this class of vector to mediate transgene expression in cells *in situ*. In good agreement with the findings *in vitro*, X-gal staining and *β*-gal immunoreactivity were restricted to the rAAV-*lacZ*-transduced explants compared with the control treatment (Figures 2(a) and 2(b), resp.), noted for at least 20 days, the longest time point examined, demonstrating transduction efficiencies of about 72–83% (Figure 2(b)).

### 3.3. Effects of the Gene Transfer via rAAV upon the Activities of Primary Human Chondrosarcoma Cells *In Vitro*

**Figure 3:**
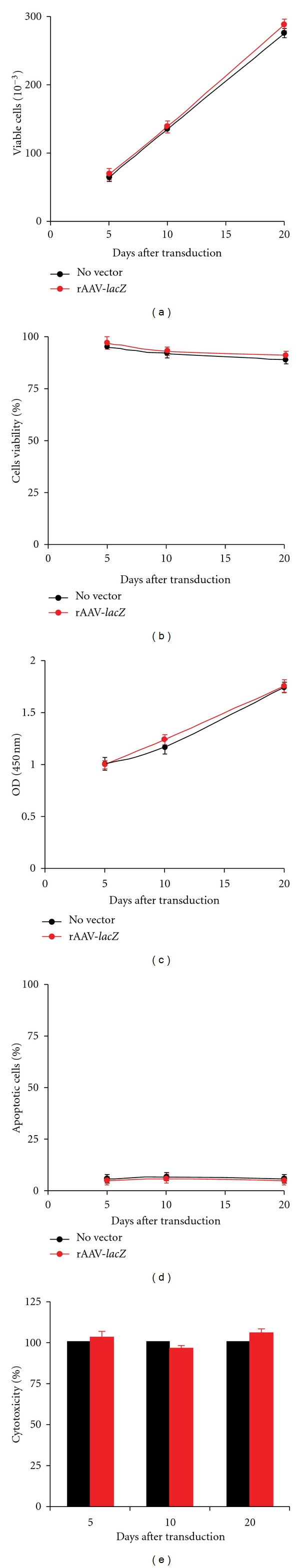
Effects of rAAV-mediated gene transfer on the survival of transduced primary human chondrosarcoma cells *in vitro*. Cells in monolayer culture were transduced as described in Figure 1 or let untreated for up to 20 days to monitor the viable cell numbers (a), cell viability (b), the rates of proliferation (WST-1 assay) (c), the levels of apoptosis (TUNEL assay) (d), and potential cytotoxic responses to the treatment by rAAV (Cytotoxicity Detection Assay LDH) (e).

Primary human chondrosarcoma cells were then transduced with the rAAV vectors in monolayer culture over time to monitor possible deleterious effects of the gene transfer procedure upon the activities of the cells *in vitro*. There was no difference between the cultures transduced with rAAV and those let untreated at any time point of the analysis for the viable cell numbers (Figure 3(a)) and percents of viability (Figure 3(b)) (*P* ≥ 0.187). Notably, the viable cell numbers significantly increased over time in both types of cultures (always *P* ≤ 0.001) [66], with stable levels of viability (always *P* ≤ 0.003). Consistent with this, there was also no difference between the rAAV-treated and untreated cells at any time point for the active rates of proliferation (WST-1 assay) (Figure 3(c)) (*P* ≥ 0.438) that also significantly increased over time (always *P* ≤ 0.005) [31]. Finally, there was no difference between the two types of cultures for the levels of apoptosis (TUNEL assay) (Figure 3(d)) (*P* ≥ 0.667) that remained expectedly low through the course of the analysis (*P* ≥ 0.347) [36, 65]. Most importantly, application of rAAV did not induce significant cytotoxic responses in transduced cells compared with the condition where the vector was not provided (Cytotoxicity Detection Kit LDH) at any time point of the analysis (Figure 3(e)) (*P* ≥ 0.150).

### 3.4. Effects of the Gene Transfer via rAAV upon the Activities of Primary Human Chondrosarcoma Cells *In Situ*

**Figure 4:**
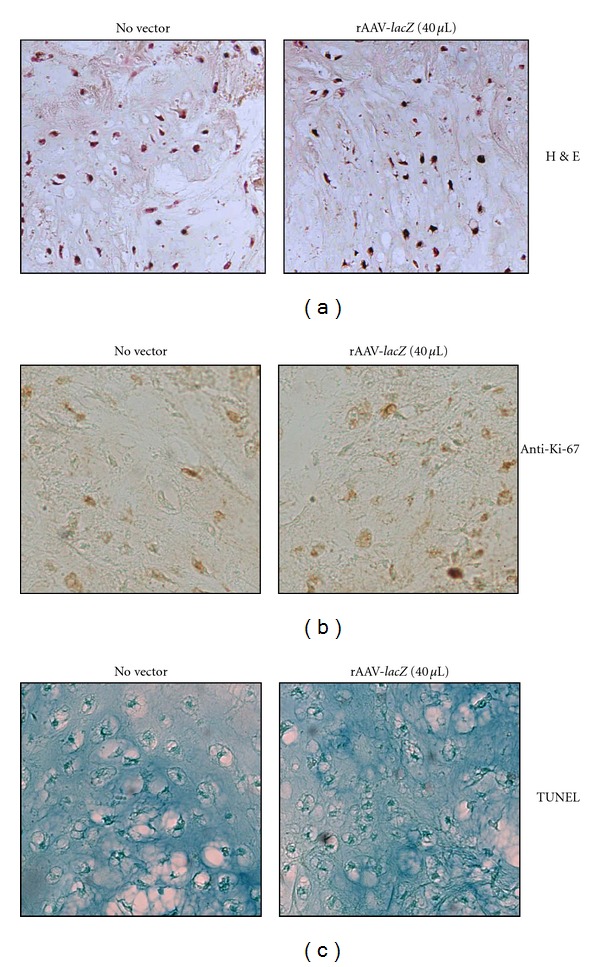
Effects of rAAV-mediated gene transfer on the survival of transduced primary human chondrosarcoma *in situ*. Human chondrosarcoma explant cultures were transduced with rAAV-*lacZ* (40 *μ*L) or let untreated and processed after 20 days for H&E staining (a), immunodetection of Ki-67 (b), and TUNEL assay (c). All at magnification ×20 except for H&E staining (magnification ×10).

**Figure 5:**
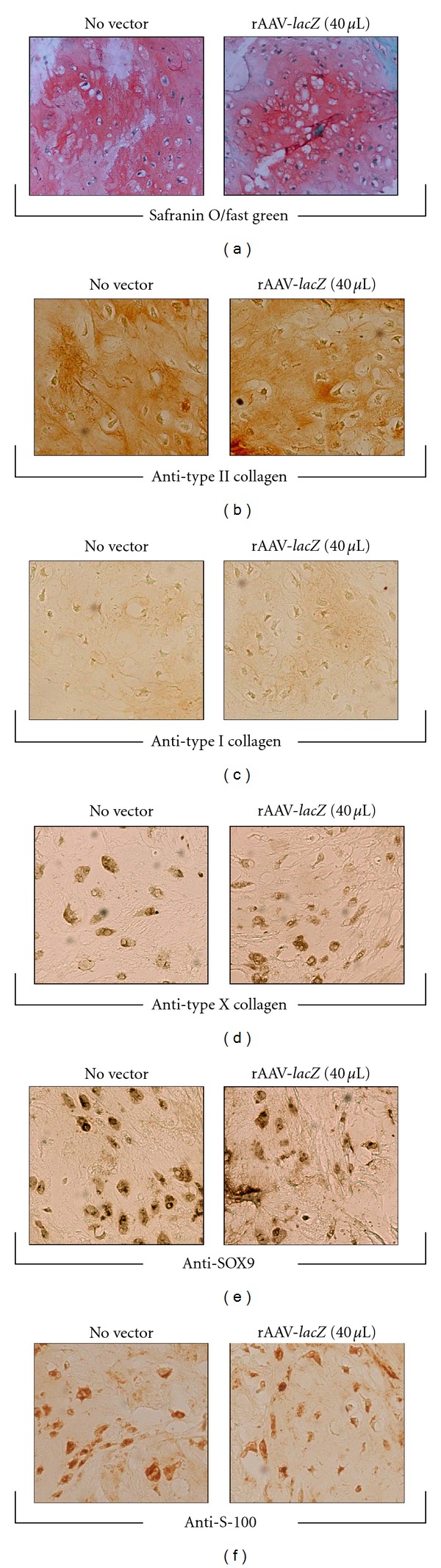
Effects of rAAV-mediated gene transfer on matrix accumulation and chondrogenic cell differentiation marker expression in transduced primary human chondrosarcoma *in situ*. Human chondrosarcoma explant cultures were transduced as described in Figure 4 and processed after 20 days for safranin O staining (a) and for immunodetection of type II collagen (b), type I collagen (c), type X collagen (d), SOX9 (e), and S-100 (f). All at magnification ×20 except for safranin O staining (magnification ×10).

The vectors were next applied for 20 days to primary human chondrosarcoma tissue explants to further evidence possible undesirable effects of the gene transfer method upon the activities of the cells *in situ*. In good agreement with the findings *in vitro*, there was no difference between the explants transduced with rAAV and those let untreated for the cell densities (H&E staining) (Figure 4(a) and Table 2) (*P* = 0.872) or for the relatively active levels of cell proliferation (Ki-67 immunodetection) (Figure 4(b) and Table 2) (*P* = 0.545) [8, 9]. There was also no difference between these explants for the levels of apoptotic cells (TUNEL assay) (Figure 4(c) and Table 2) (*P* = 0.667) that remained low during the time of evaluation [67]. When the presence of major extracellular matrix components was estimated by histological, immunohistochemical, and histomorphometric analyses, again no difference could be evidenced between rAAV-treated and untreated explants for the strong intensities of safranin O staining (Figure 5(a) and Table 2) (*P* = 0.545) and of type II collagen immunostaining (a marker of differentiated chondrocytes) (Figure 5(b) and Table 2) (*P* = 0.659) [5–9, 14]. Application of rAAV also did not modify the relatively low levels of type I collagen expression compared with the controls (Figure 5(c) and Table 2) (*P* = 0.290) [6–8]. There was also no difference between the explants for the percents of cells that deposited type X collagen (a marker of hypertrophic chondrocytes) (Figure 5(d) and Table 2) (*P* = 0.684) or stained positive for SOX9 (master chondrogenic transcription factor) (Figure 5(e) and Table 2) (*P* = 0.784) [5, 7, 8, 14–16]. Finally, an evaluation of S-100 expression revealed that application of rAAV did not influence the high expression levels of this chondrocytic differentiation marker (Figure 5(f) and Table 2) (*P* = 0.108) [5–7, 16]. 

## 4. Discussion

Approaches based on the direct application of candidate gene sequences might provide strong tools for an effective, durable treatment of chondrosarcoma as the conventional options used currently do not allow for simple and functional therapies that block the progression of the tumor in patients. To achieve this goal, classical gene vehicles such as nonviral, adenoviral, retroviral and lentiviral vectors have been employed with relatively moderate success so far due to low-gene transfer efficiencies, immunogenicity and toxicity, and the risk of insertional mutagenesis [19, 25–30, 32, 36, 40, 41, 45, 46]. In marked contrast, the use of vectors based on the replication-defective human adenoassociated virus (AAV) might be better suited as recombinant AAV (rAAV) has been shown to transduce most of the cells of human origin very efficiently *in vitro*, *in situ*, but most remarkably directly *in vivo* over extended periods of time (as a result of the maintenance of the transgenes under stable episomal forms), without activating significant host-immune responses (due to the removal of viral gene sequences in the recombinant genome) [48–50]. Yet, to our best knowledge this class of vectors has not yet been tested for its ability to target directly human chondrosarcoma in a safe and effective manner.

In the present study, we therefore administered various marker gene constructs to examine the permissivity of primary human chondrosarcoma cells and tissue to direct application of rAAV vectors and evaluated the safety of the approach *in vitro* and *in situ* by different evaluation methods. We show that our various vectors can mediate elevated and prolonged levels of transgene expression in these cells in both systems tested (primary monolayer and tissue explant cultures), probably due to the ability of these small vectors to penetrate the extracellular matrix *in situ* [58, 59, 61]. Specifically, the data indicate that continuous, efficient, and sustained expression of the transgenes was noted for at least 20 days following vector application both *in vitro* and *in situ* with transduction efficiencies reaching 72–90%, consistent with findings where rAAV was used to target various other cells of the musculoskeletal system (articular chondrocytes, meniscal fibrochondrocytes, mesenchymal stem cells) [54, 60, 61], probably due to the well-maintained numbers of vector copies present in transduced cells (3–5 copies per cell over time). Absolute transduction of cells was not achieved, here, but it is important to note that we used relatively low MOIs (20–40) compared with other experimental settings [68, 69], and 100% efficacy may have been attained at higher vector doses. This is in marked contrast with previous reports demonstrating lower efficacy and shorter periods of transgene expression (between some hours and some days) using nonviral, adenoviral, retroviral, or lentiviral vectors [26–30, 32, 33, 40, 41, 47] unless the genetically modified cells are selected for extended evaluation setups (nonviral and retroviral vectors) [25, 29, 36, 41, 45, 46].

Most importantly, and in view of a safe use of rAAV in future experimental or clinical settings *in vivo*, we provide evidence that administration of this class of vector has no undesirable effects on the activities of primary human chondrosarcoma cells both *in vitro* and *in situ* by assessing the rates of cell viability and proliferation (expectedly high but not increased following transduction), the occurrence of apoptotic events (always low to absent), and the levels of metabolic processes (significant but unaffected by rAAV), as well as the potential cytotoxic responses to the gene transfer method (undetectable). Furthermore, no events of recombinant viral genome integration could be demonstrated in cultures over the time of evaluation. The next reasonable step of the current approach will be to select a strong therapeutic candidate for cloning in our vectors, evaluate its efficacy and duration of gene expression in human chondrosarcoma cells (primary and tissue cultures) but also in a relevant animal model *in vivo* [19, 41], and subsequently investigate the potency of the vector against cell expansion, differentiation, and transformation, as well as tumor progression and invasiveness [19, 25–30, 32, 40, 41, 45, 46]. Among the different factors tested so far in the form of a genetic sequence, agents like the HSV-tk or OSM have been reported for their antitumor, proapoptotic properties *in vitro* and *in vivo* [19, 28, 41]. Still, only partial and/or transient effects were noted with these components [19, 41], although this might have resulted from the moderate efficiency of the gene vehicles themselves (adenoviral and retroviral vectors there) *vis-à-vis* rAAV. Also noteworthiy, excessive candidate (OSM) gene activity occurred in some cases, leading to the death of several OSM-treated animals whereas control animals survived [19]. Other agents with possibly less aggressive properties may be further considered, as various studies reported the protective effects of p53 and targeted siRNAs among others, against chondrosarcoma cell death, growth inhibition, and invasiveness *in vitro* [25–27, 29, 30, 32, 36, 40, 45, 46]. Interestingly, such sequences may be provided simultaneously as separate rAAV can be conveniently applied at the same time to their targets with effective expression of the cotransgenes [58, 70].

In summary, the results of this study indicate that direct application of rAAV vectors can promote long-term, efficient, and safe transgene expression in primary human chondrosarcoma cells both *in vitro* and most notably *in situ*, providing further motivation to develop rAAV-based therapeutic gene treatments to be tested over extended periods of time for the treatment of human chondrosarcoma.

## Figures and Tables

**Table 1:** Luciferase activity in transduced human chondrosarcoma cells *in vitro*.

Vector (40 *μ*L)	Day 5	Day 10	Day 20
rAAV-*lacZ *	6.8 (0.1)	5.4 (0.1)	3.5 (0.2)
rAAV-*luc *	24.7 (1.3)^a^	13.8 (0.5)^a^	89.7 (3.4)^a^

Activity is given in RLU/*μ*g total proteins. Values are given as mean of all cultures (SD). ^a^Statistically significant *vis-à-vis* control (rAAV-*lacZ*) treatment.

**Table 2:** Histomorphometric analyses in transduced human chondrosarcoma cells *in situ* (day 20).

Assay	No vector	rAAV-*lacZ *
Cell densities (cells/mm^2^)	394 (6)	401 (8)
Ki-67 staining (%)	52 (3)	54 (4)
TUNEL staining (%)	2 (1)	1 (1)
Matrix staining (%)	85 (2)	83 (3)
Type II collagen staining (pixels)	79 (3)	80 (4)
Type I collagen staining (pixels)	8 (2)	10 (3)
Type X collagen staining (%)	79 (3)	77 (4)
SOX9 staining (%)	84 (2)	83 (3)
S-100 staining (%)	82 (3)	81 (2)

Values are given as mean of all cultures (SD).
